# Supplementary material for: Structure and activation of the human autophagy-initiating ULK1C:PI3KC3-C1 supercomplex
Source: Nat Struct Mol Biol. 2025 May 29;32(9):1596–605. doi: 10.1038/s41594-025-01557-x (PMC12440827; doi:10.1038/s41594-025-01557-x)
Supplement: Supplementary file 1 — Reporting Summary [file 41594_2025_1557_MOESM1_ESM.pdf]

nature portfolio

Corresponding author(s): James H. Hurley

Last updated by author(s): Mar 18, 2025

Reporting Summary

Nature Portfolio wishes to improve the reproducibility of the work that we publish. This form provides structure for consistency and transparency in reporting. For further information on Nature Portfolio policies, see our Editorial Policies and the Editorial Policy Checklist.

Statistics

For all statistical analyses, confirm that the following items are present in the figure legend, table legend, main text, or Methods section.

n/a	Confirmed	
☐	☒	The exact sample size (*n*) for each experimental group/condition, given as a discrete number and unit of measurement
☐	☒	A statement on whether measurements were taken from distinct samples or whether the same sample was measured repeatedly
☒	☐	The statistical test(s) used AND whether they are one- or two-sided<br>*Only common tests should be described solely by name; describe more complex techniques in the Methods section.*
☒	☐	A description of all covariates tested
☒	☐	A description of any assumptions or corrections, such as tests of normality and adjustment for multiple comparisons
☐	☒	A full description of the statistical parameters including central tendency (e.g. means) or other basic estimates (e.g. regression coefficient) AND variation (e.g. standard deviation) or associated estimates of uncertainty (e.g. confidence intervals)
☐	☒	For null hypothesis testing, the test statistic (e.g. *F*, *t*, *r*) with confidence intervals, effect sizes, degrees of freedom and *P* value noted<br>*Give P values as exact values whenever suitable.*
☒	☐	For Bayesian analysis, information on the choice of priors and Markov chain Monte Carlo settings
☒	☐	For hierarchical and complex designs, identification of the appropriate level for tests and full reporting of outcomes
☒	☐	Estimates of effect sizes (e.g. Cohen's *d*, Pearson's *r*), indicating how they were calculated

Our web collection on statistics for biologists contains articles on many of the points above.

Software and code

Policy information about availability of computer code

Data collection	SerialEM (ver. 4.0.20), ASTRA (ver. 5.3.4) , ImageLab (ver. 5.2.1, DiscoverMP (ver. 2024R2)
Data analysis	CryoSPARC (v4.1), Topaz (0.2.5a), ChimeraX (ver 1.5, EMAN2 (ver 2.0), ISOLDE (ver 1.5), Phenix (1.20.1,) AlphaFold (Ver. 2), coot (ver. 0.9.8), ColabFold (ver 1.3.0), MMseqs2 (ver. 13-45111).

For manuscripts utilizing custom algorithms or software that are central to the research but not yet described in published literature, software must be made available to editors and reviewers. We strongly encourage code deposition in a community repository (e.g. GitHub). See the Nature Portfolio guidelines for submitting code & software for further information.

Data

Policy information about availability of data

All manuscripts must include a data availability statement. This statement should provide the following information, where applicable:

- Accession codes, unique identifiers, or web links for publicly available datasets
- A description of any restrictions on data availability
- For clinical datasets or third party data, please ensure that the statement adheres to our policy

The cryo-EM maps were deposited in the Electron Microscopy Data Bank (EMDB) under accession codes EMD-40658 (ULK1C (2:1:1) core), EMD-45297 (ULK1C:PI3KC3-C1 supercomplex), EMD-40715 (ULK1C (2:2:2) core in the PI3KC3-C1 mixture), and EMD-40735 (ULK1C (2:2:2) core of the ATG13450-517 truncation mutant). The structural coordinates were deposited in the Protein Data Bank (PDB) under accession codes PDBID:8SOI (ULK1C (2:1:1) core), PDBID:9C82

(ULK1C:PI3KC3-C1 supercomplex), PDBID:8SQZ (ULK1C (2:2:2) core in the PI3KC3-C1 mixture), and PDBID:8SRM (ULK1C (2:2:2) core of the ATG13450-517 truncation mutant). Protocols were deposited in protocols.io (Plasmid construction (DOI: 10.17504/protocols.io.bp2l6x3b5lqe/v1), Sample preparation for cryo-EM samples of FIP200NTD:ATG13(363-517)-ULK1MIT complexes (DOI: 10.17504/protocols.io.e6nvwjxw7lmk/v1), Strep pull-down assay (DOI: 10.17504/protocols.io.3byl4jmpolo5/v1), GST pull down assay (DOI: 10.17504/protocols.io.36wgqj2xxvk5/v1), Microscopy-based GSH bead protein-protein interaction assay (DOI: 10.17504/protocols.io.4r3l27xdxg1y/v1), Size Exclusion Chromatography with Multiangle Light Scattering (SEC-MALS) (DOI: 10.17504/protocols.io.j8nlkom4xv5r/v1), Sample preparation of FIP200:PI3KC3-C1 complex for cryo-EM (DOI: 10.17504/protocols.io.5qpvorjezv4o/v1), Image processing and 3D reconstruction (DOI: 10.17504/protocols.io.x54v9d99mg3e/v1), Model building, validation, and visualization (DOI: 10.17504/protocols.io.j8nlkw77wl5r/v1), Sample vitrification and cryo-EM data acquisition (DOI: 10.17504/protocols.io.kqdg39rreg25/v1)). Generation of CRISPR constructs (DOI: 10.17504/protocols.io.j8nlkkzo6l5r/v1), Mass photometry (DOI: 10.17504/protocols.io.kqdg3keq7v25/v2). Raw data files for gel scans were uploaded to Zenodo (DOI: 10.5281/zenodo.10056244). Source data files for mass photometry were uploaded to Zenodo (DOI: 10.5281/zenodo.15047085). Plasmids developed for this study were deposited at Addgene.org. See Extended Data Table 1 for complete details.

Research involving human participants, their data, or biological material

Policy information about studies with human participants or human data. See also policy information about sex, gender (identity/presentation), and sexual orientation and race, ethnicity and racism.

Reporting on sex and gender	N/A
Reporting on race, ethnicity, or other socially relevant groupings	N/A
Population characteristics	N/A
Recruitment	N/A
Ethics oversight	N/A

Note that full information on the approval of the study protocol must also be provided in the manuscript.

Field-specific reporting

Please select the one below that is the best fit for your research. If you are not sure, read the appropriate sections before making your selection.

☒ Life sciences ☐ Behavioural & social sciences ☐ Ecological, evolutionary & environmental sciences

For a reference copy of the document with all sections, see nature.com/documents/nr-reporting-summary-flat.pdf

Life sciences study design

All studies must disclose on these points even when the disclosure is negative.

Sample size	For cryo-EM, enough particles were collected to achieve necessary resolution to answer the biological question, as is standard in the field. Cell experiments were performed at least as three replicates as stated in the figure legend, according to current practices in the field. Statistical analysis was performed on experiments for which the sample size included at least 3 biological replicates. Sample sizes were based on previous experience and current standards in the field.
Data exclusions	No data were excluded from analyses
Replication	Each cryo-EM grid square was collected on once because the holes can only be collected on once. This is standard in the field. Each PAGE gel was repeated two to four times, which was mentioned in figure legends individually. Cell experiments from Figures 2i-2l were repeated 3 times as provided in the figure legend.
Randomization	Randomization is not applicable to this study.
Blinding	Blinding is not relevant for this study because the experimental conditions must be known to interpret the results.

Reporting for specific materials, systems and methods

We require information from authors about some types of materials, experimental systems and methods used in many studies. Here, indicate whether each material, system or method listed is relevant to your study. If you are not sure if a list item applies to your research, read the appropriate section before selecting a response.

Materials & experimental systems

n/a	Involved in the study
☐	☒ Antibodies
☐	☒ Eukaryotic cell lines
☒	☐ Palaeontology and archaeology
☒	☐ Animals and other organisms
☒	☐ Clinical data
☒	☐ Dual use research of concern
☒	☐ Plants

Methods

n/a	Involved in the study
☒	☐ ChIP-seq
☒	☐ Flow cytometry
☒	☐ MRI-based neuroimaging

Antibodies

Antibodies used

Anti-VCP (Cell Signaling Technology, Cat# 2649, RRID:AB_2214629, 1:1000), Mouse anti-Actin (Cell Signaling Technology, Cat# 4967S, RRID: AB_330288, 1:1000), Rabbit anti-ATG13 (Cell Signaling Technology, Cat# 7613, RRID: AB_10827645, 1:1000), Rabbit anti-ATG14(S29) (Cell Signaling Technology, Cat# 92340S, RRID: AB_2800182, 1:1000), Rabbit anti-ATG14 (Cell Signaling Technology, Cat# 96752S, RRID: AB_2737056, 1:1000), Rabbit anti-FIP200 (Cell Signaling Technology, Cat# 12436S, RRID: AB_2797913, 1:1000), HRP anti-Flag antibody (Abcam, Cat# b49763, RRID:AB_869428, 1:1000), Donkey anti-goat IgG (Cy5) (Abcam, Cat# ab6566, RRID:AB_955056, 1:300), Anti-GST Antibody (Cytiva, Washington DC, Cat# 27457701, RRID:AB_771432, 1:2000), Rabbit anti-MBP antibody (Thermo Fisher Scientific, Cat# PA1-989, RRID:AB_559988, 1:10000), Alexa Fluor 488 goat anti-Riabbit IgG (Thermo Fisher Scientific, Cat# A11034, RRID:AB_2576217, 1:500), Anti-HALO (Promega, Cat# G9211, RRID:AB_2688011, 1:1000).

Validation

For all antibodies used in this study were commercially available. Validation statement for each primary antibody is provided on the manufacture's website: Anti-VCP (Cell Signaling Technology, Cat# 2649): https://www.cellsignal.com/products/primary-antibodies/vcp-7f3-rabbit-mab/2649?srsltid=AfmBOoq4tLBfkpHlr9kvTEwdz8YtTlRJLL4P6SwY0C-ZnwUn07MJbP6l, Validated for WB and cited by 16 publications. Mouse anti-Actin (Cell Signaling Technology, Cat# 4967S): https://www.cellsignal.com/products/primary-antibodies/b-actin-antibody/4967?srsltid=AfmBOopLogEoaBCdOcHv666-eP9SmxBvAxe5V_Q4xTWOAb5PosGBlyqD. Validated for WB and cited by 3824 publications. Rabbit anti-ATG13 (Cell Signaling Technology, Cat# 7613): https://www.cellsignal.com/products/primary-antibodies/atg4a-d62c10-rabbit-mab/7613?srsltid=AfmBOor204sYTb3pG87Rag-1Wtwigzxg8OCIjO_D7-g0EpoyJTd_ZuEk. Validated for WB, IP and cited by 19 publications. Rabbit anti-ATG14(S29) (Cell Signaling Technology, Cat# 92340S): https://www.cellsignal.com/products/primary-antibodies/phospho-atg14-ser29-d4b8m-rabbit-mab/92340?srsltid=AfmBOoo3AW_a5y7F5Z0XDmTIG4kqQxJ4sFQvliD3ULKI3rblhXYs7nLd. Validated for WB, IF, F and cited by 23 publications. Rabbit anti-ATG14 (Cell Signaling Technology, Cat# 96752S): https://www.cellsignal.com/products/primary-antibodies/atg14-d1a1n-rabbit-mab/96752?srsltid=AfmBOor7DmnMWAsLf60VgV83dft3ZQrmagWHMGDow6wJ-fboLw4NNmVe. Validated for WB, IP and cited by 52 publications. Rabbit anti-FIP200 (Cell Signaling Technology, Cat# 12436S): https://www.cellsignal.com/products/primary-antibodies/fip200-d10d11-rabbit-mab/12436?srsltid=AfmBOoqhJ2s2zL4bTCTIi5bOsfPcOuEMBmKcTiFdB6phY4Ky3X9hwVpC. Validated for WB, IP and cited by 109 publications. HRP anti-Flag antibody (Abcam, Cat# b49763): https://www.abcam.com/en-us/products/primary-antibodies/hrp-ddddk-tag-binds-to-flag-tag-sequence-antibody-m2-ab49763?srsltid=AfmBOopiZ8XsS8G9AmaGtlndjKZ5NneaglYJrycjfEAYPdh98mIlB2vO. Validated for WB, ELISA and cited by over 85 publications. Donkey anti-goat IgG (Cy5) (Abcam, Cat# ab6566): https://www.abcam.com/en-us/products/secondary-antibodies/donkey-goat-igg-h-l-cy5-preadsorbed-ab6566?srsltid=AfmBOorvfsJG_O6TLDYUxlHzGeZq_SPdTLdTyoF263eElmnBi0barBTJ. Validated for WB and cited by 42 publications. Anti-GST Antibody (Cytiva, Washington DC, Cat# 27457701):https://www.cytivalifesciences.com/en/us/shop/chromatography/resins/affinity-tagged-protein/anti-gst-antibody-p-06000?srsltid=AfmBOoqIE2SS7jBtMby1gpFd0L0zbUIidO1toBETpcMuX73CQu2cpwMJ. Cited by 213 publications. Rabbit anti-MBP antibody (Thermo Fisher Scientific, Cat# PA1-989): https://www.thermofisher.com/antibody/product/Maltose-Binding-Protein-Antibody-Polyclonal/PA1-989. Validated for WB and cited by 9 publications. Alexa Fluor 488 goat anti-Riabbit IgG (Thermo Fisher Scientific, Cat# A11034): https://www.thermofisher.com/antibody/product/Goat-anti-Rabbit-IgG-H-L-Highly-Cross-Adsorbed-Secondary-Antibody-Polyclonal/A-11034. Validated for ICC/IF and cited by 7504 publications. Anti-HALO (Promega, Cat# G9211): https://www.promega.com/products/protein-detection/primary-and-secondary-antibodies/anti-halotag-monoclonal-antibody/?catNum=G9211. Validated for IF and cited by 114 publications.

Eukaryotic cell lines

Policy information about cell lines and Sex and Gender in Research

Cell line source(s)

HEK293 GnTi cells were provided by the Cell Culture Facility of University of California, Berkeley. HeLa knockout cell lines generated in this study was submitted to Cellosaurus.

Authentication

All cell lines were validated by morphological analysis and routinely tested for absence of mycoplasma

Mycoplasma contamination

All cells were routinely tested for mycoplasma contamination using MycoAlert Mycoplasma Detection kit (Lonza, LT07-318). All cell lines were negative throughout the study.

Commonly misidentified lines
(See ICLAC register)

No cell lines used in this study were found in the database of commonly misidentified cell lines that is maintained by ICLAC and NCB BioSample.

Plants

Seed stocks	N/A
Novel plant genotypes	N/A
Authentication	N/A
