# Supplementary figures and images for: Structure and activation of the human autophagy-initiating ULK1C:PI3KC3-C1 supercomplex
Source: Nat Struct Mol Biol. 2025 May 29;32(9):1596–605. doi: 10.1038/s41594-025-01557-x (PMC12440827; doi:10.1038/s41594-025-01557-x)

Figure 1d

Coomassie

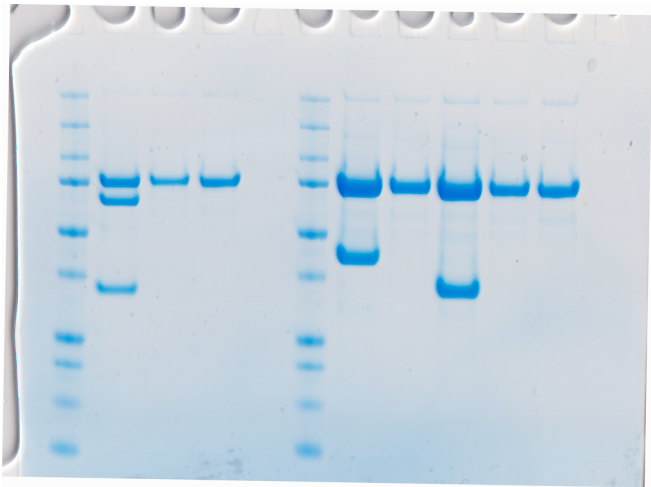

Flag

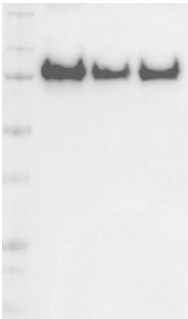

MBP

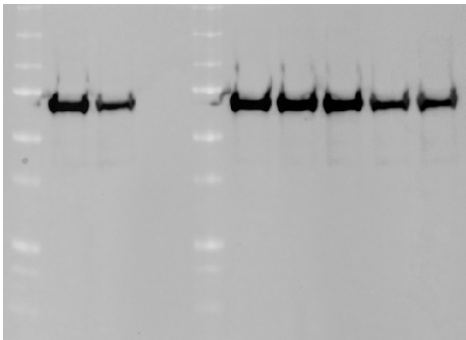

GST

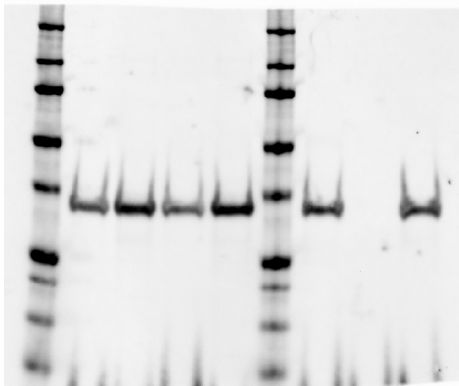

Figure 1e

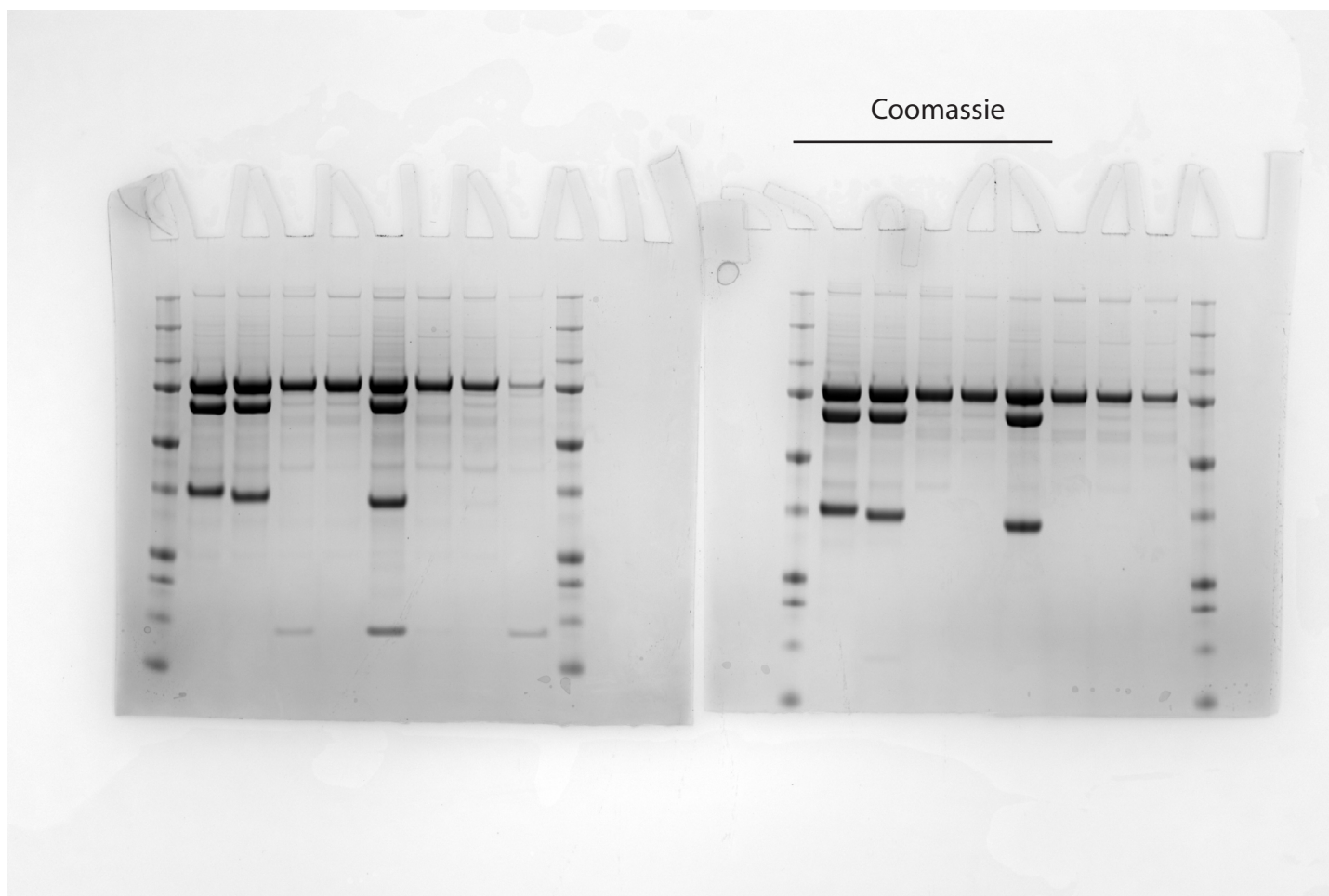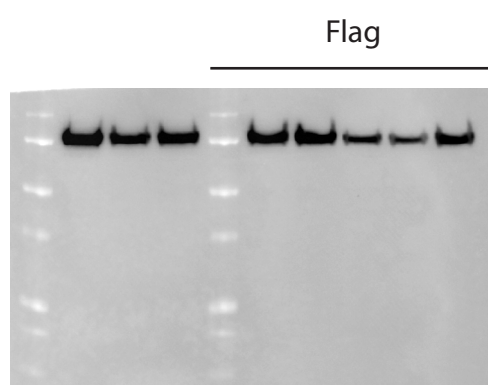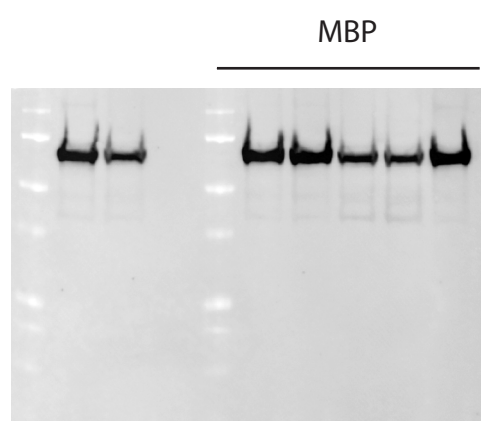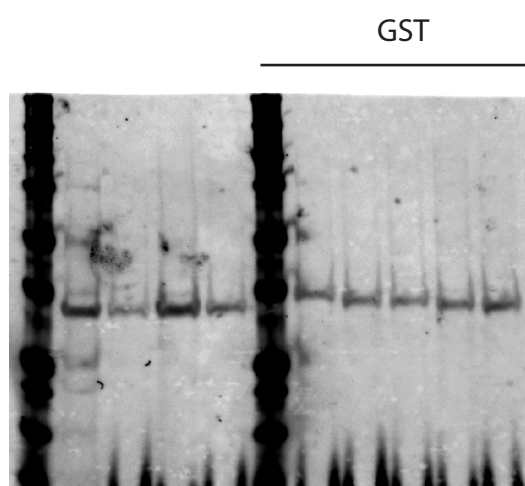

Figure 1i

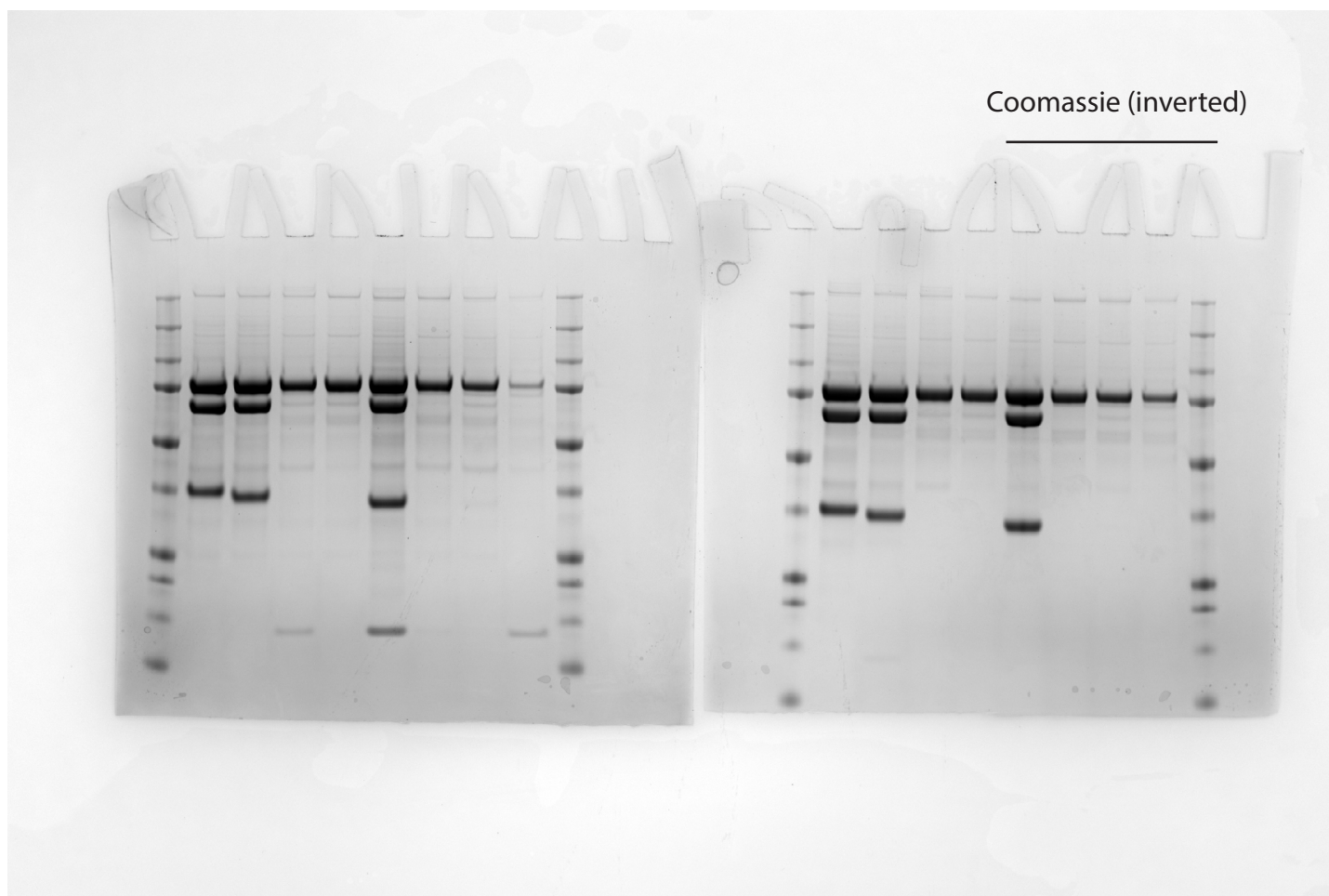

Flag

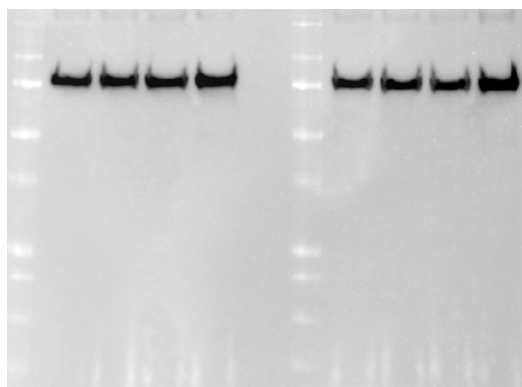

MBP

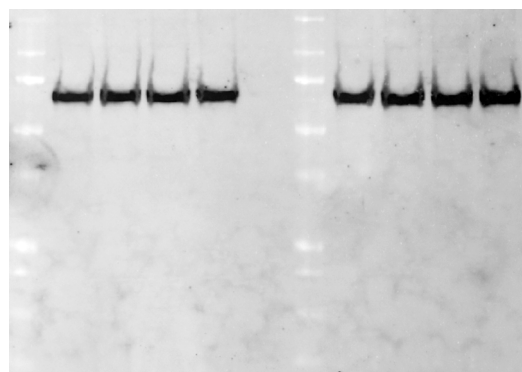

GST

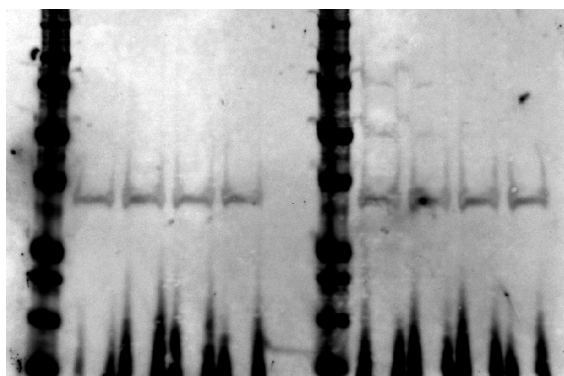

Supplement: Supplementary file 4 — Unprocessed Coomassie blue-stained gels and western blots. [file 41594_2025_1557_MOESM4_ESM.pdf]

Figure 2c

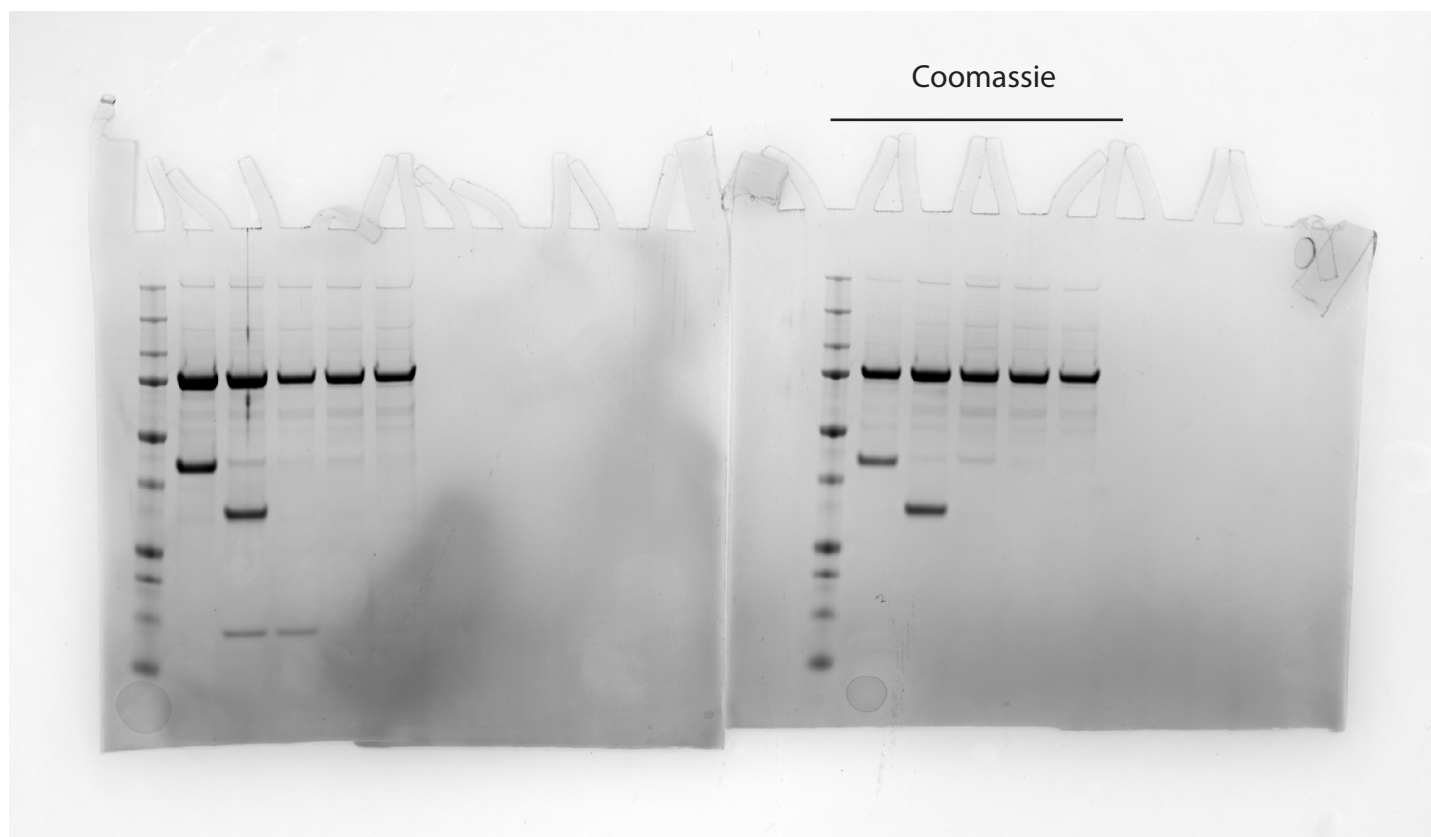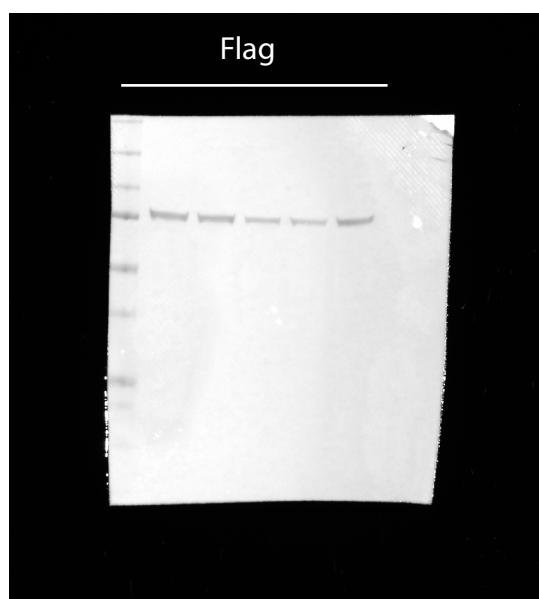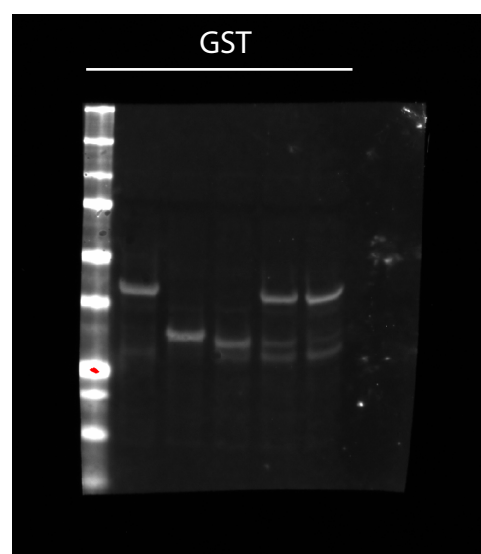

Figure 2f

Coomassie

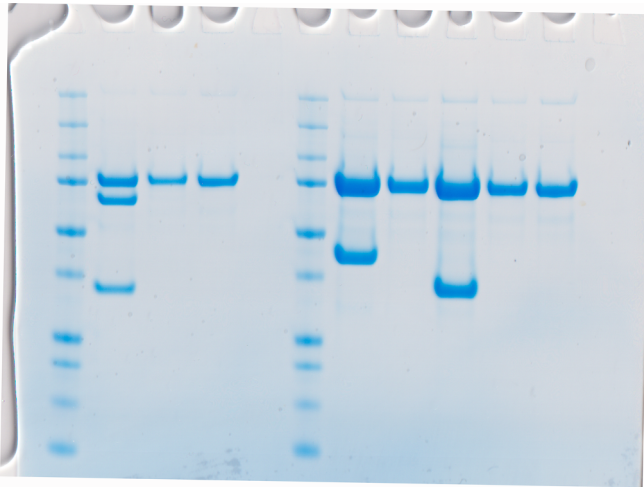

Flag

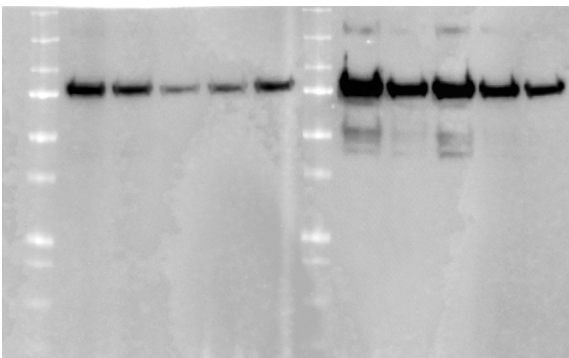

GST

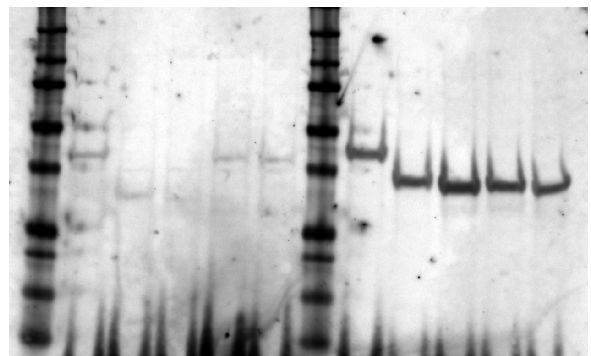

Figure 2i

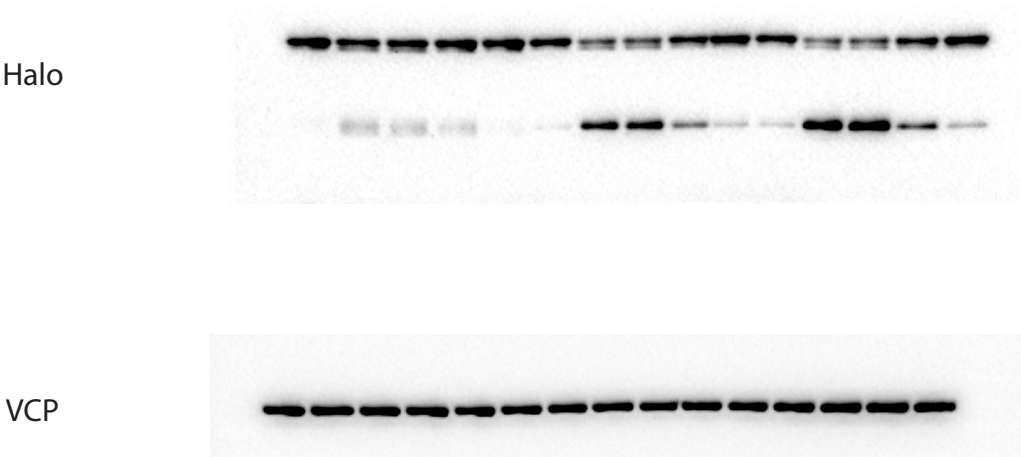

Figure 2k

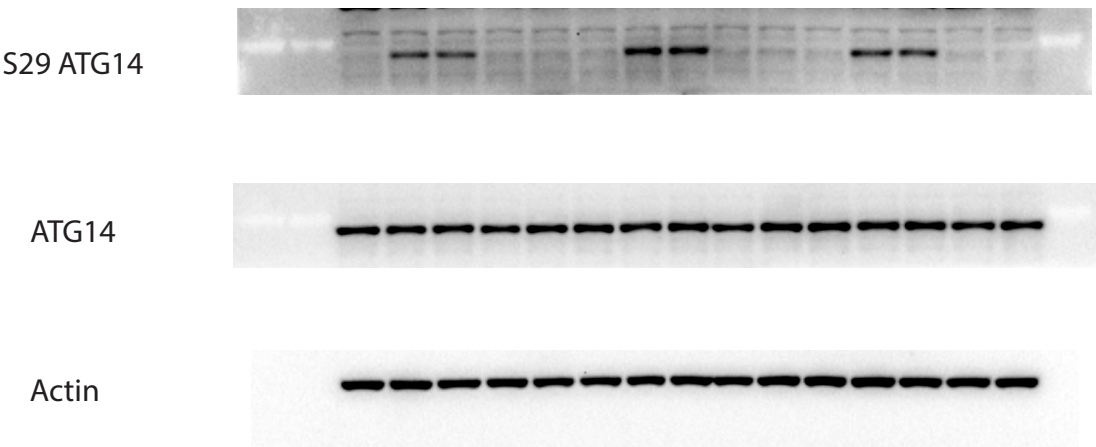

Supplement: Supplementary file 5 — Unprocessed Coomassie blue-stained gels and western blots. [file 41594_2025_1557_MOESM5_ESM.pdf]

Figure 3c

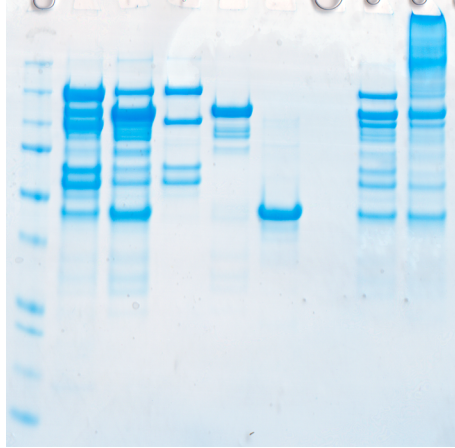

Supplement: Supplementary file 6 — Unprocessed Coomassie blue-stained gel. [file 41594_2025_1557_MOESM6_ESM.pdf]

Figure 4a

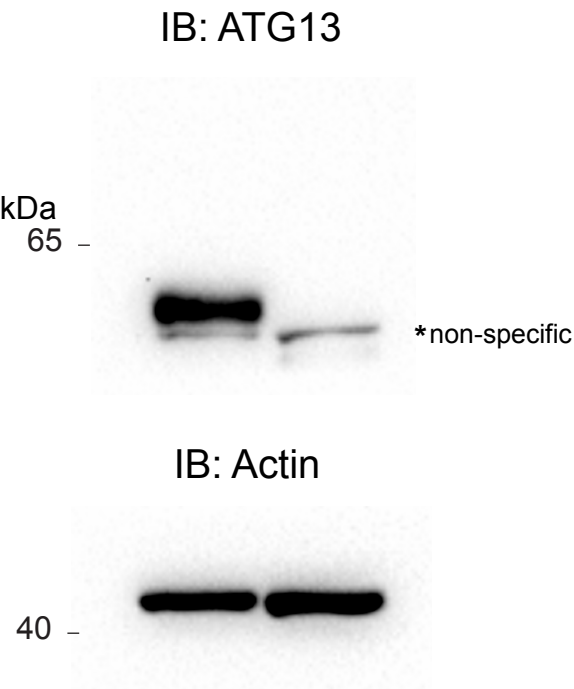

Figure 4b

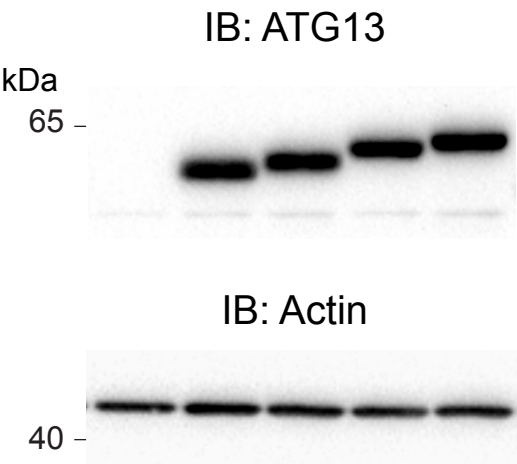

Supplement: Supplementary file 9 — Unprocessed western blots. [file 41594_2025_1557_MOESM9_ESM.pdf]
